# Kinetic Characterization of Cerium and Gallium Ions as Inhibitors of Cysteine Cathepsins L, K, and S

**DOI:** 10.3390/ijms23168993

**Published:** 2022-08-12

**Authors:** Marko Novinec, Primož Bembič, Milica Janković, Marija Kisilak, Jakob Kljun, Iztok Turel

**Affiliations:** Faculty of Chemistry and Chemical Technology, Department of Chemistry and Biochemistry, University of Ljubljana, Večna pot 113, 1000 Ljubljana, Slovenia

**Keywords:** protease, kinetic mechanism, zinc, lead

## Abstract

Heavy metal ions can disrupt biological functions via multiple molecular mechanisms, including inhibition of enzymes. We investigate the interactions of human papain-like cysteine endopeptidases cathepsins L, K, and S with gallium and cerium ions, which are associated with medical applications. We compare these results with zinc and lead, which are known to inhibit thiol enzymes. We show that Ga^3+^, Ce^3+^, and Ce^4+^ ions inhibit all tested peptidases with inhibition constants in the low micromolar range (between 0.5 µM and 10 µM) which is comparable to Zn^2+^ ions, whereas inhibition constants of Pb^2+^ ions are one order of magnitude higher (30 µM to 150 µM). All tested ions are linear specific inhibitors of cathepsin L, but cathepsins K and S are inhibited by Ga^3+^, Ce^3+^, and Ce^4+^ ions via hyperbolic inhibition mechanisms. This indicates a mode of interaction different from that of Zn^2+^ and Pb^2+^ ions, which act as linear specific inhibitors of all peptidases. All ions also inhibit the degradation of insoluble elastin, which is a common target of these peptidases in various inflammatory diseases. Our results suggest that these ions and their compounds have the potential to be used as cysteine cathepsin inhibitors in vitro and possibly in vivo.

## 1. Introduction

Cysteine cathepsins are important players in lysosomal and extracellular protein turnover in humans and other animals [1,2]. As the name implies, they are peptidases from the cysteine class, specifically the papain-like family. In humans, there are eleven paralogs that differ in their substrate specificity, endo-/exoproteolytic mode of action, and expression patterns. In this work, we investigated cathepsins L, K, and S. All are closely related members of the cathepsin L-like subgroup of papain-like peptidases that act as endopeptidases [3]. Cathepsin L is ubiquitously expressed and has recently received much attention as one of the peptidases involved in the entry of SARS-CoV-2 into the cell [4]. Both knockdown and inhibition of this peptidase can significantly reduce the infectivity of SARS-CoV-2 [5,6]. Cathepsins K and S are expressed in tissue-specific patterns in accordance with their specific functions. Cathepsin K is a central peptidase in bone turnover [7], whereas cathepsin S plays an important role in antigen presentation [8]. Both are also considered potential drug targets in diseases related to their specific biological functions. Cathepsin K is a promising target for the treatment of osteoporosis [9]. Its inhibitor odanacatib (Merck & Co., New York, NY, USA) was evaluated in a phase 3 clinical trial but unfortunately had to be discontinued due to rare but significant side effects [10]. Nevertheless, cathepsin K remains a viable target in osteoporosis [11]. In addition, the inhibitor MIV-711 (Medivir, Huddinge, Sweden) shows promise for the treatment of osteoarthritis [12]. Cathepsin S, on the other hand, is a target for the treatment of inflammatory diseases, such as rheumatoid arthritis and pulmonary fibrosis, and several cathepsin S inhibitors, e.g., petesicatib (Roche, Basel, Switzerland), have shown potential in clinical trials [13,14]. However, as with cathepsin K, no inhibitor has yet been approved for medical use.

Many metal ions are vital to living organisms when present in small amounts [15]. On the other hand, some are also highly cytotoxic [16] and the degree of their toxicity depends on the ligands bound to the metal ion. For example, the toxicity of Cu^2+^ has recently been associated with its detrimental effect on the tricarboxylic acid cycle, a central metabolic pathway in aerobic organisms [17]. Several common heavy metal ions, such as Zn^2+^, Fe^3+^, and Cu^2+^ are known inhibitors of thiol enzymes due to their direct interaction with catalytic cysteine residues [18]. Zn^2+^, in particular, plays a well-known role in the control of lysosomal cysteine peptidases [19]. Importantly, Zn^2+^ has recently been characterized as a potent inhibitor of the SARS-CoV-2 main protease and viral replication [20]; interestingly, we have also recently discovered that zinc pyrithione exhibits potent in vitro inhibitory activity against SARS-CoV-2 papain-like protease as well as human cathepsin L [21]. Other metal ions are considerably less hazardous, and some are now commonly used for medical purposes. Gallium-68 is a widely used isotope for medical imaging [22] and Ga^3+^ ions have no significant toxic effects on cell growth and metabolism [23]. Cerium oxide CeO_2_ is commonly used in nanocomposite grafts and has the added advantage of scavenging free radicals [24]. Cerium ions can exist as either Ce^3+^ or Ce^4+^ and shuttling between the two oxidation states is thought to underlie the antioxidant properties of such Ce-containing nanoparticles [25]. Both forms are cytotoxic only at high concentrations (in the range of ~10^−4^ M) [26,27,28], but CeO_2_ nanoparticles have been found to potentially cause DNA damage [29].

To date, no studies have systematically investigated the effects of gallium or cerium ions on thiol enzymes. Here, we investigate the molecular mechanisms by which Ga^3+^, Ce^3+^_,_ and Ce^4+^ ions inhibit selected cysteine endopeptidases of the papain-like family (cathepsins K, L, and S) and compare them with Zn^2+^ and Pb^2+^ ions. We focus in detail on their kinetic modes of action and their effects on the hydrolysis of the protein substrate bovine neck ligament elastin.

## 2. Results

### 2.1. Spectroscopic Analysis of Interaction between Ions and Substrate

**Figure 1:**
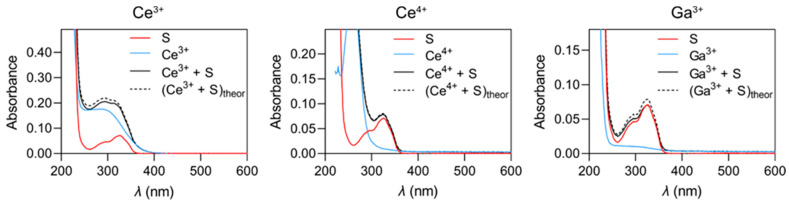
Absorption spectra of Ce^3+^, Ce^4+^, and Ga^3+^ salts in the presence and absence of the synthetic substrate Z-Phe-Arg-AMC. Spectra were recorded in 50 mM sodium acetate buffer pH 5.5 in a quartz cuvette with an optical path of 1 cm. The concentrations of Ce^4+^ and Ga^3+^ ions in the experiments shown were 0.5 mM and the concentration of Ce^3+^ ions was 0.05 mM. The concentration of the substrate was 5 µM.

Pearson’s principle of hard-soft acid–base theory (HSAB) is a simple rule for predicting the stability of complexes formed between metal ions and ligands. Undoubtedly, it is a very useful concept, but we must be aware that it also has some limitations (e.g., it is very general and has no direct quantitative scale for Lewis acid-base strength; treatment of ambidentate reactivity; many exceptions occur, etc.) [30,31,32,33]. Of the ions tested, Zn^2+^ and Pb^2+^ are considered intermediate (borderline) acids, while Ce^3+^, Ce^4+^, and Ga^3+^ ions are hard acids [31,34]. Cerium ions have already been shown to form complexes with amino acids and dipeptides [35,36]. Therefore, we first determined whether Ce^3+^, Ce^4+^, and Ga^3+^ ions interacted with the synthetic peptide substrates used in the study or otherwise interfered with the kinetic measurements. We measured the UV-Vis absorption spectra of a representative substrate (Z-Phe-Arg-AMC) in the presence and absence of the metal ions studied (specifically, their salts Ga(NO_3_)_3_, CeCl_3_, and (NH_4_)_2_Ce(NO_3_)_6_, respectively) in reaction buffer (Figure 1). The substrate produced a broad peak with a maximum at 325 nm and a shoulder at 295 nm. The spectra of the Ga^3+^ and Ce^4+^ salts at a concentration of 0.5 mM, which is comparable to the highest concentrations used in the kinetic experiments, produced signals only below 350 nm, while CeCl_3_ absorbed much stronger below 400 nm. Therefore, the spectra of CeCl_3_ had to be recorded at a significantly lower concentration (0.05 mM) to obtain a signal intensity comparable to the substrate. Since the excitation wavelength used in the kinetic experiments was 370 nm, all progress curves obtained in the presence of CeCl_3_ were corrected to account for the inner filter effect [37]. The spectra of the individual salts mixed with the substrate showed no significant shifts in maxima or deviations in intensity from the theoretical sums of the salt and the substrate alone, leading us to conclude that there are no significant interactions or spectral phenomena under these conditions that would interfere with the kinetic measurements of enzyme activity other than correction for the inner filter effect.

Certain cerium complexes have been shown to possess hydrolase activity [38,39]. Therefore, we also investigated whether the cerium and gallium salts had detectable hydrolytic activity toward the substrate Z-Phe-Arg-AMC at a metal ion concentration of 0.5 mM, the highest concentration used in the kinetic experiments. No such activity was detected, indicating that the salts themselves do not generate background activity in the kinetic experiments.

### 2.2. Kinetic Mechanisms of Action

Metal ions, which according to the HSAB theory are soft or intermediate Lewis acids, are known inhibitors of thiol enzymes interacting with the thiol group of the active site catalytic cysteine residue, which is itself a soft Lewis base [34]. Kinetically, the interaction with the catalytic cysteine residue implies a linear specific (competitive) inhibition mechanism in which the presence of the metal ion prevents the binding of the substrate in the active site. Examples of such ions include Zn^2+^ and Pb^2+^ ions, both of which are intermediate Lewis acids and are known to interact with cysteine peptidases [18,19]. However, Ce^3+^, Ce^4+^, and Ga^3+^ ions, which are the focus of our study, are hard Lewis acids, and therefore, could affect the activity of cysteine peptidases via other kinetic mechanisms. Therefore, we characterized these interactions using appropriate synthetic substrates to measure and compare the activity of cathepsins L, K, and S in the presence and absence of metal ions. As sources of the individual ions, we used their salt forms CeCl_3_, (NH_4_)_2_Ce(NO_3_)_6_ and Ga(NO_3_)_3_, respectively. Although the counterions in these salts are not identical (Cl^−^ vs. NO_3_^−^) this is of no practical consequence because these anions were never found to affect the activity of the investigated peptidases at such low concentrations (up to 2 mM). Since the reactions were carried out in 0.1 M sodium acetate buffer, their contribution to the total ionic strength of the reaction mixture was also minimal.

Titration curves in the presence of increasing concentrations of ions were performed first and then complemented with specific velocity plots [40] to diagnose the mechanisms of interaction. The kinetic modification parameters of all cathepsin/metal ion pairs characterized in this work are summarized in Table 1. Our results showed that the above assumptions held true overall. Regarding their activity, the tested ions can be distinguished by two different properties. The first subdivision is between linear (full) and hyperbolic (partial) inhibitors; the second is the distinction between ions acting in a 1:1 stoichiometry and those acting via more complex mechanisms that likely involve the binding of more than one metal ion to the peptidase. Mechanisms with a 1:1 stoichiometry were adequately described using the general modifier mechanism [41], while others were fitted using the four-parameter logistic equation to obtain estimates for their *EC*_50_ values. Interestingly, the effects of each ion also differed between the peptidases, which are all closely related in terms of structure, catalytic properties, and substrate specificity [42]. Most significantly, cathepsin L was inhibited by all ions via a linear specific mechanism, whereas cathepsins K and S were inhibited by Ce^3+^, Ce^4+^, and Ga^3+^ ions via hyperbolic mechanisms.

**Figure 2:**
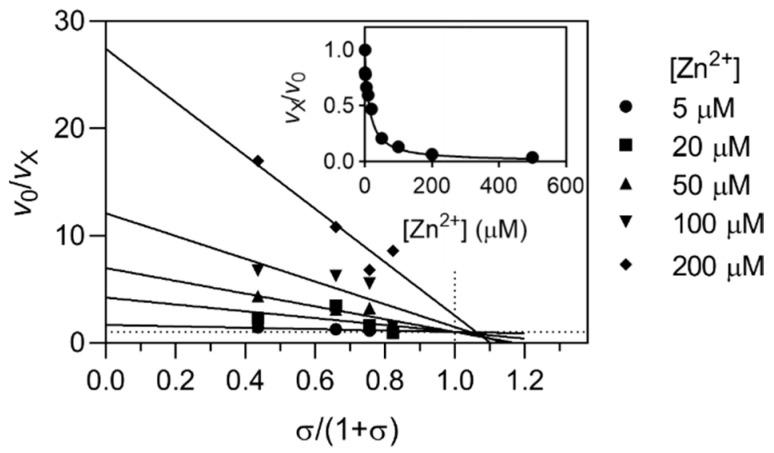
Specific inhibition of cathepsin K by Zn^2+^ ions. The mechanism was determined by the specific velocity plot using the fluorogenic substrate Z-Phe-Arg-AMC. *σ* is equal to [S]/*K*_m_. An additional titration curve recorded with 5 µM substrate (*σ* = 1) is shown in the inset. Experiments were performed at 25 °C in 0.1 M sodium acetate buffer pH 5.5.

As expected, Zn^2+^ and Pb^2+^ ions acted as linear specific inhibitors binding in a 1:1 stoichiometry (Figure 2 and Supplementary Figures S1 and S2). Values of the equilibrium dissociation constants (i.e., inhibition constants, a measure of the binding affinity) for the binding of Zn^2+^ ions to the target peptidases were in the range of 10^−6^ M for all peptidases tested, whereas the binding of Pb^2+^ was weaker (between 2.6 × 10^−5^ and 1.4 × 10^−4^ M).

**Figure 3:**
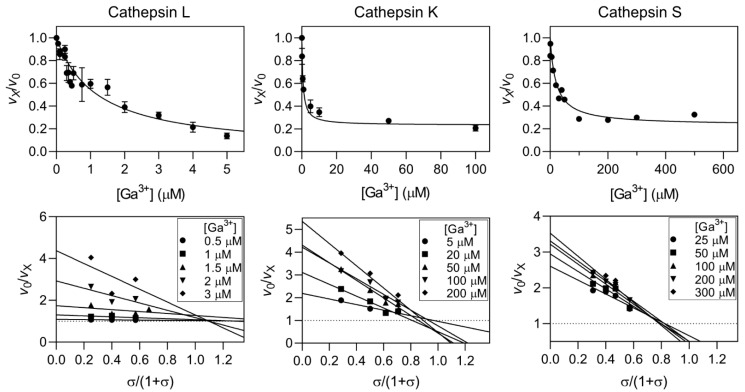
Inhibition of cathepsins L, K, and S by Ga^3+^ ions. The titration curves are shown in the upper panels and the specific velocity plots used to determine the inhibition mechanisms are shown in the lower panels. Activity was measured with synthetic fluorogenic substrates (Z-Leu-Arg-AMC, Z-Phe-Arg-AMC and Z-Val-Val-Arg-AMC for cathepsins L, K, and S, respectively) at 25 °C in 0.1 M sodium acetate buffer pH 5.5. *σ* is equal to [S]/*K*_m_. The error bars in the titration curves represent the S.E.M. of multiple independent experiments.

**Figure 4:**
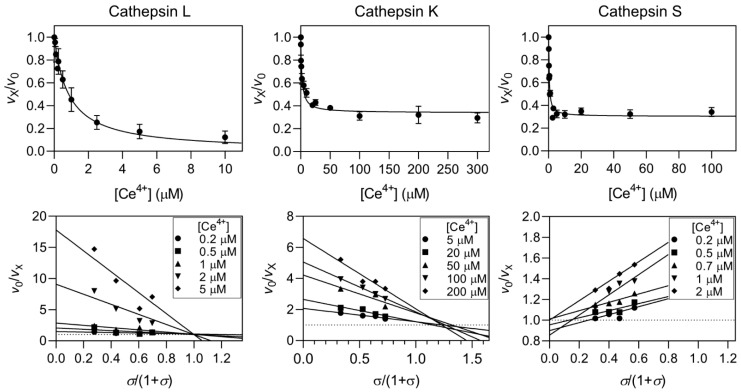
Inhibition of cathepsins L, K, and S by Ce^4+^ ions. The titration curves are shown in the upper panels and the specific velocity plots used to determine the inhibition mechanisms are shown in the lower panels. Activity was measured with synthetic fluorogenic substrates (Z-Leu-Arg-AMC, Z-Phe-Arg-AMC and Z-Val-Val-Arg-AMC for cathepsins L, K, and S, respectively) at 25 °C in 0.1 M sodium acetate buffer pH 5.5. *σ* is equal to [S]/*K*_m_. The error bars in the titration curves represent the S.E.M. of multiple independent experiments.

Ga^3+^ ions acted as linear inhibitors of cathepsin L but as hyperbolic inhibitors of cathepsins K and S, leaving about 20% of residual enzyme activity at saturation (Figure 3). The determined kinetic mechanism in the case of cathepsin L was linear specific inhibition, i.e., it affected enzyme activity in the same way as Zn^2+^ and Pb^2+^ ions. The kinetic mechanisms for the interaction with cathepsins K and S were diagnosed as hyperbolic mixed inhibition, trending towards activation at high substrate concentrations. Similar mechanisms were previously diagnosed for small molecule modifiers of cathepsins K and S, which presumably act through allosteric mechanisms [43,44,45], suggesting that this is a common mechanism by which these peptidases may be regulated by chemically diverse modifiers. Furthermore, the affinity of Ga^3+^ ions for cathepsin L was one order of magnitude higher than for the remaining two (*K*_X_ values of 6 × 10^−7^ M for cathepsin L versus ~4 × 10^−6^ M for cathepsins K and S). The observed trend continued with Ce^4+^ ions which acted as linear specific inhibitors of cathepsin L and hyperbolic inhibitors of cathepsins K and S (Figure 4 and Table 1). The inhibition mechanisms were diagnosed as hyperbolic mixed in the case of cathepsin K and hyperbolic catalytic in the case of cathepsin S. Again, an affinity for cathepsin L was several-fold higher than for cathepsins K and S (*K*_X_ values of 0.4 × 10^−7^ M for cathepsin L versus ~1.2 × 10^−6^ M for cathepsins K and S).

**Figure 5:**
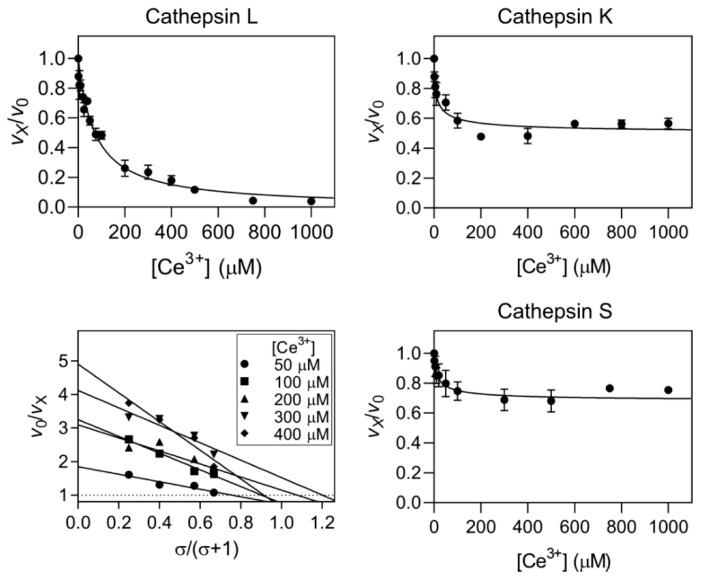
Inhibition of cathepsins L, K, and S by Ce^3+^ ions. For cathepsin L, the titration curve is shown in the upper panel and the specific velocity plot is shown in the lower panel. For cathepsins K and S, only the titration curves are shown. Activity was measured using synthetic fluorogenic substrates (Z-Leu-Arg-AMC, Z-Phe-Arg-AMC and Z-Val-Val-Arg-AMC for cathepsins L, K, and S, respectively) at 25 °C in 0.1 M sodium acetate buffer pH 5.5. *σ* is equal to [S]/*K*_m_. The error bars in the titration curves represent the S.E.M. of multiple independent experiments.

All the interactions described above could be fitted with mechanisms assuming a 1:1 binding stoichiometry. In contrast, Ce^3+^ ions again acted as linear specific inhibitors of cathepsin L, but titrations of cathepsins K and S yielded kinetic profiles that deviated from “classical” models with 1:1 stoichiometry (Figure 5). These profiles were fitted using the four-parameter logistic equation, where the value of the coefficient *h* (equivalent to the Hill coefficient) indicates the deviation from regular 1:1 stoichiometry and the value of *EC*_50_ is an estimate of the overall affinity of the interaction (Table 1). The binding affinity of Ce^3+^ ions for all peptidases was significantly lower than that of Ce^4+^ ions (*K*_X_ and *EC*_50_ values of ~1 × 10^−5^ M, respectively). In addition, the extent of inhibition of cathepsins K and S at saturation was the weakest of all metal ions, resulting in residual enzyme activity of approximately 60% and 70%, respectively. It should be noted that such behavior is neither unprecedented nor unexpected. Similar profiles were obtained, for example, for the inhibition of human leukocyte elastase by glycosaminoglycans, which the authors attributed to several possible binding modes between the two partners [46]. In this case, the structural basis for this behavior remains to be determined. However, given that metal ions can interact with the side chains of various amino acid residues, interaction with multiple sites on the enzymes is a plausible possibility.

### 2.3. Hydrolysis of Bovine Neck Ligament Elastin

**Figure 6:**
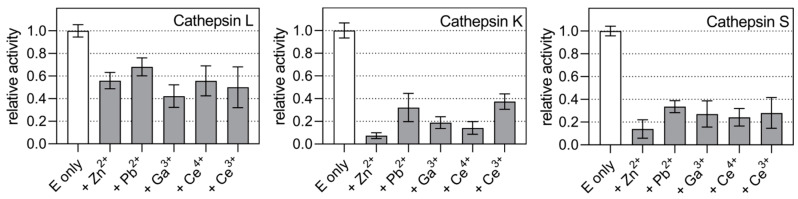
Inhibition of bovine neck ligament elastin degradation by selected metal ions. Plots show relative enzyme activities normalized to the activities of each cathepsin in the absence of metal ions. Final concentrations of metal ions and peptidases in the reaction mixtures were 0.2 µM and 0.5 mM, respectively. The concentrations of the degradation products were measured after their derivatization with fluorescamine, as described previously [51]. The error bars represent the S.E.M. of at least three replicates.

Synthetic substrates are much smaller than natural protein substrates, and although they are convenient for characterizing the kinetic properties of enzymes, the results obtained with their use are only an approximation of the proteolytic activity on protein substrates. In our previous work with hyperbolic, presumably allosteric, modifiers of cathepsin K we observed that these small molecules were often significantly less effective when protein substrates were used [44]. To complement the results obtained in the previous section and evaluate the efficacy of the tested metal ions in this context, we used bovine neck ligament elastin as a protein substrate. Elastin is an insoluble, structural protein that is often the target of cysteine cathepsins in pathological conditions [47,48,49,50] and we have previously studied the mechanisms by which cathepsins L, K, and S degrade this insoluble material [51]. All metal ions were tested at 0.5 mM concentration, thus assuming saturation of peptidases with the ions based on the kinetic experiments in the previous section. The results in Figure 6 show that cathepsin L was most resistant to inhibition. Its activity was reduced by all metal ions to between 50% and 70% of the uninhibited enzyme. Ga^3+^ ions were the most potent inhibitor, but differences between the ions were not significant. In contrast, Zn^2+^ ions were the strongest inhibitor of elastin degradation by cathepsins K and S. All ions reduced the elastinolytic activity to between 10% and 40% of the uninhibited enzyme. Thus, the results obtained for cathepsins K and S corresponded well to the residual activities observed in assays with synthetic substrates. The exception was Ce^3+^ ions, which were significantly more potent than in assays with synthetic substrates. Elastin degradation by cathepsin K and S has been proposed to involve exosites on the peptidases [52,53]. It is, therefore, possible that the binding of Ce^3+^ ions (and others) to these exosites contributes to their inhibitory effect.

## 3. Discussion

The detrimental effects of heavy metal ions on many enzymes are well-known. However, the details of many of these interactions remain poorly understood. We show that selected metal ions, namely Ce^3+^, Ce^4+^, and Ga^3+^, can inhibit human cysteine cathepsin endopeptidases by different kinetic mechanisms than the specific inhibition observed for ions interacting with catalytic cysteines, e.g., Zn^2+^ and Pb^2+^ [18,19]. Although the structural basis for these observations remains to be elucidated, given their relatively low cytotoxicity, this approach may offer new opportunities for targeting these enzymes in vitro and in vivo, either by the ions alone or, more feasibly, in complexes with synthetic inhibitors of these peptidases. To date, numerous chemically diverse inhibitors of cysteine cathepsins have been investigated that achieved high selectivity for their targets as well as high affinity with *K*_X_ values in the nanomolar range [54,55]. However, in the absence of clinically approved inhibitors, further efforts are needed to develop alternative targeting strategies. The affinity of the metal ions tested here is much lower than that of the most advanced inhibitors and they are also expected to interact nonspecifically with many other protein targets in vivo, making their use alone not feasible. However, the formation of defined metal complexes with suitable ligands might result in inhibitors with higher affinity and selectivity. Often, metal complexes are not stable under physiological conditions. A famous example of such complexes is the ruthenium complex NAMI-A with anticancer activity [56]. The instability of the complexes can lead to the release of free metal ions from the complexes which can then act synergistically with other synthetic inhibitors. Therefore, it is also important to study the interactions of free metal ions with their potential biological targets, such as cysteine cathepsins.

In addition, these ions provide new opportunities for studying the structural basis for hyperbolic inhibition of papain-like peptidases and the potentially allosteric mechanisms underlying this kinetic behavior. Furthermore, the size of some of these ions allows for the coordination of multiple enzyme molecules to the same ion. This has previously been observed in a crystal structure of cathepsin K (PDB accession code 3C9E), where the binding of a Ca^2+^ ion results in a symmetric cathepsin K dimer [57]. In any case, detailed structural characterization will be required to obtain definitive answers.

A peculiar finding from our experiments is that the inhibition mechanisms differ markedly between cathepsin L and the other two. This suggests binding to different sites on the peptidases and/or different responses of the peptidases to the binding of these ions. Cathepsins K and S are more closely related to each other than to cathepsin L [1], but all three have similar substrate specificity [42]. This finding does, however, complement the fact that hyperbolic inhibitors have been described for cathepsins K and S [43,44,45], whereas no such inhibitors are known for cathepsin L. It is possible that cathepsin L is incapable of such modification due to a much more limited activity space, although it has a similar conformational space to cathepsins K and S [58]. Therefore, an allosteric mode of action of Ce^3+^, Ce^4+^, and Ga^3+^ ions cannot be excluded and possibly involves interactions of Ce^3+^ ions with multiple sites on cathepsins K and S, as discussed in Section 2.2

Cathepsin L also behaved differently from cathepsins K and S in elastinolytic assays (Figure 6). These differences could be due to their different kinetic profiles of interaction with the metal ions and/or differences in their interactions with elastin. We have previously shown that cathepsin L adheres much less efficiently to the insoluble substrate than cathepsins K and S [51]. In the same work, we also observed that some proteinaceous inhibitors were poorly efficient in preventing elastin degradation [51]. In any case, this observation reiterates the fact that the efficiency of peptidase inhibitors can also depend on the substrate. It should also be noted that much longer incubation times were required for these assays than for the kinetic experiments. Therefore, it is possible that the oxidation states of some metal ions changed during incubation. Cerium ions in particular are known to undergo redox reactions in aqueous solutions [59,60]. Other effects, such as hydration and hydrolysis of metal ions can also affect their interaction with peptidases [61]. However, it can also be expected that such changes occur in vivo, so these assays approximate in vivo processes in this regard as well. Of course, the physiological concentrations of these metals are much lower than the concentrations used in our experiments. Cerium is present in body fluids in sub-nanomolar concentrations [62], which is much lower than the determined binding constants. Therefore, it is unlikely to affect cathepsin activity. Gallium concentrations in blood are in the micromolar range [63] which is comparable to the *K*_X_ values determined for cathepsins L, K, and S (Table 1). However, most of the gallium was bound to erythrocytes, which limits its availability, and together with the fact that it can interact with many different target proteins, its specific effect on cathepsins is also unlikely. In comparison, the amount of Ga-68 used for radiolabeling is extremely small (a few pmol) [22], and therefore, unlikely to affect cysteine peptidase activity.

Overall, this study shows that metal ions, such as cerium and gallium can affect the activity of cysteine peptidases to varying degrees and by different mechanisms, and it is among the first detailed studies of the kinetic mechanisms of such interactions. Together with the prospect of developing novel metal complexes for use as inhibitors of these peptidases, it identifies cerium and gallium ions as research tools for in vitro studies of the mechanisms of hyperbolic inhibition and will serve as a basis for further exploration of the interactions between peptidases and metal ions.

## 4. Materials and Methods

### 4.1. Materials

Ga(NO_3_)_3_, CeCl_3_, and (NH_4_)_2_Ce(NO_3_)_6_ were purchased from Honeywell (Charlotte, NC, USA). All synthetic substrates were from Bachem (Bubendorf, Switzerland). Powdered bovine neck ligament elastin was from the Elastin Products Company, Inc. (Owensville, MO, USA). Recombinant human cathepsins L, K, and S were produced as zymogens in *E. coli* and activated in vitro, as described previously [45,64]. Active enzyme concentrations were determined by active site titration with the irreversible inhibitor E-64 (Bachem, Bubendorf, Switzerland).

### 4.2. UV-Vis Spectroscopy

UV-Vis spectra of Ga(NO_3_)_3_, CeCl_3_, and (NH_4_)_2_Ce(NO_3_)_6_ were recorded in 50 mM sodium acetate buffer pH 5.5 in a 1 mL quartz cuvette with an optical path of 1 cm at room temperature on a Varian Cary 50 UV-Vis spectrometer at a scan speed setting “slow”. The concentrations of Ce^4+^ and Ga^3+^ were 0.5 mM and the concentration of Ce^3+^ was 0.05 mM. The spectra were recorded in the presence and absence of Z-Phe-Arg-AMC (benzyloxycarbonyl-Phe-Arg-7-amido-4methylcoumarin), one of the substrates used in kinetic measurements. The final concentration of the substrate was 5 µM. Background spectra of buffer alone were recorded separately and subtracted automatically from the samples.

### 4.3. Enzyme Kinetics

Kinetic measurements were performed with recombinant human cathepsins at final concentrations in the 0.2 nM to 2 nM range. Reactions were performed in 0.1 M sodium acetate buffer pH 5.5 in single-use acrylic cuvettes (1 cm × 1 cm). Hydrolysis of all synthetic substrates was monitored fluorimetrically at an excitation wavelength λ_ex_ = 370 nm and emission wavelength λ_em_ = 455 nm. The specific substrates used were Z-Leu-Arg-AMC for cathepsin L, Z-Phe-Arg-AMC for cathepsin K, and Z-Val-Val-Arg-AMC for cathepsin S. Reducing agents (e.g., DTT) were omitted from the reaction mixture to prevent complex formation with metal ions. Enzyme stock solutions were kept on ice and regularly tested to assure no loss of activity during each assay.

Initial reaction rates were determined from the slopes of the progress curves and analyzed in GraphPad Prism software version 9.3 (GraphPad Software, La Jolla, CA, USA) using appropriate kinetic models, as described below.

### 4.4. Elastin Degradation Assays

Degradation of insoluble elastin was performed and analyzed according to the protocol in ref. [51]. Briefly, reactions were performed using 200 µL samples, each containing 1 mg of insoluble bovine neck ligament elastin. The enzymes were added to a final concentration of 0.2 µM and the metal ions to a final concentration of 0.5 mM. Samples were incubated at 37 °C for 120 min on an Eppendorf Thermomixer with shaking at 1200 rpm, and the reactions were then stopped by the addition of trichloroacetic acid to a final concentration of 5% (*w*/*V*). Samples were cleared by centrifugation and 100 µL of the supernatant was mixed with 1400 µL of 0.2 M sodium borate buffer and 500 µL of a 0.15 mg/mL solution of fluorescamine in acetone. The fluorescence of the samples was measured at an excitation wavelength of λ_ex_ = 390 nm and emission wavelength of λ_em_ = 480 nm. Blank samples without added enzyme were run in parallel and their fluorescence values were subtracted from the samples. Experiments were performed in three replicates of each sample and each experiment was repeated multiple times to assure reproducibility of the results.

### 4.5. Kinetic Models

**Scheme 1:**
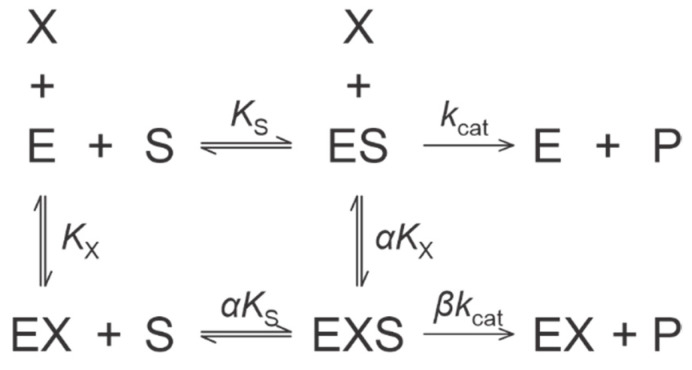
General modifier mechanism for the interaction between enzyme (E), substrate (S) and modifier (X).

All experimental data were processed and analyzed with GraphPad Prism 9.3 (GraphPad Software, La Jolla, CA, USA). The same software was also used to generate all graphical representations of the data. To characterize the kinetic mechanisms of all metal ions (X) that are bound in a 1:1 stoichiometry, we used the general modifier mechanism [41] shown in Scheme 1.

In this model, the reaction rate in the presence of modifier X is defined as:

(1) $$v_{X}=\frac{v_{0}\times \left(1+\sigma \right)\times \left(1+\beta \times \frac{\left[X\right]}{{\alpha \times K}_{X}}\right)}{1+\frac{\left[X\right]}{K_{X}}+\sigma \times \left(1+\frac{\left[X\right]}{{\alpha \times K}_{X}}\right)}\;\;$$

where *v*_0_ is the reaction rate in the absence of an inhibitor, *K*_X_ is the equilibrium dissociation constant of complex EX and *σ* is equal to [S]/*K*_m_. Dimensionless coefficients *α* and *β* describe the effects of X on substrate affinity (*α)* and catalytic rate (*β*) of the enzyme, respectively. Together, both coefficients define the kinetic mechanism of the inhibitor.

To diagnose the mechanism, we used the specific velocity plot [40]. Specific velocity *v*_0_/*v*_X_ is defined as:

(2) $$\frac{v_{0}}{v_{X}}=\frac{\left[X\right]\times \left(\frac{1}{{\alpha \times K}_{X}}-\frac{1}{K_{X}}\right)}{1+\beta \times \frac{\left[X\right]}{{\alpha \times K}_{X}}}\times \frac{\sigma }{1+\sigma }+\frac{1+\frac{\left[X\right]}{K_{X}}}{1+\beta \times \frac{\left[X\right]}{{\alpha \times K}_{X}}}\;\;$$

The plot of *v*_0_/*v*_X_ against *σ*/(1 + *σ*) produces straight lines that always intersect at *v*_0_/*v*_X_ = 1. The *x*-value (*σ*/(1 + *σ*)) of the intersection depends on the kinetic mechanism and is used to diagnose the latter. Values of parameters *α*, *β* and *K*_X_ can be determined from replots, obtained by plotting the values of the straight lines extrapolated to *σ*/(1 + *σ*) = 0 (a) and *σ*/(1 + *σ*) = 1 (b) versus 1/[X] as defined below:

(3) $$\frac{a}{a-1}=\frac{{\alpha \times K}_{X}}{\alpha -\beta }\times \frac{1}{\left[X\right]}+\frac{\alpha }{\alpha -\beta }$$

(4) $$\frac{b}{b-1}=\frac{{\alpha \times K}_{X}}{1-\beta }\times \frac{1}{\left[X\right]}+\frac{1}{1-\beta }$$

The effect of metal ions which could not be described by the above model was analyzed using a modified form of the four-parameter logistic equation adapted to enzyme inhibition:

(5) $$v_{X}{=v}_{0}-\frac{\left(v_{0}-v_{\infty }\right)\times {\left[X\right]}^{h}}{{\mathrm{EC}}_{50}{}^{h}+{\left[X\right]}^{h}}$$

where *v*_∞_ is the residual reaction rate at saturation, *EC*_50_ is the metal ion concentration necessary to achieve a half-maximal effect, i.e., midway between *v*_0_ and *v*_∞_, and *h* is a dimensionless coefficient (equivalent to the Hill coefficient) used to identify deviation from 1:1 binding stoichiometry when *h* ≠ 1. The value of *EC*_50_ is used here as an empirical comparison term for assessing metal ion efficiency towards different enzymes when the kinetic mechanism cannot be sufficiently described by the general modifier mechanism.

## Figures and Tables

**Table 1:** Kinetic parameters for the inhibition of cysteine cathepsins L, K, and S by selected metal ions.

Ion	Enzyme	Mechanism	*K*_X_ (µM)	*α*	*β*
Zn^2+^	Cathepsin L	Linear specific	7.5 ± 0.9		
	Cathepsin K	Linear specific	7.3 ± 0.3		
	Cathepsin S	Linear specific	2.9 ± 0.5		
Pb^2+^	Cathepsin L	Linear specific	26 ± 12		
	Cathepsin K	Linear specific	135 ± 13		
	Cathepsin S	Linear specific	50 ± 5		
Ga^3+^	Cathepsin L	Linear specific	0.60 ± 0.04		
	Cathepsin K	Hyperbolic mixed	4.1 ± 0.8	11.5 ± 1.0	2.4 ± 1.4
	Cathepsin S	Hyperbolic mixed	3.7 ± 0.6	11.3 ± 1.0	3.3 ± 0.5
Ce^4+^	Cathepsin L	Linear specific	0.42 ± 0.03		
	Cathepsin K	Hyperbolic mixed	1.3 ± 0.2	3.3 ± 0.6	0.73 ± 0.04
	Cathepsin S	Hyperbolic catalytic	1.2 ± 0.2	0.18 ± 0.01	0.18 ± 0.01
Ce^3+^	Cathepsin L	Linear specific	12 ± 6		
**Ion**	**Enzyme**	**Mechanism**	***EC*_50_ (µM)**	***h***	
Ce^3+^	Cathepsin K	Hyperbolic	11 ± 6	0.6 ± 0.2	
	Cathepsin S	Hyperbolic	17 ± 5	0.7 ± 0.1	

## Data Availability

Data are contained within the article or Supplementary Material.

## Supplementary Materials

The following supporting information can be downloaded at: https://www.mdpi.com/article/10.3390/ijms23168993/s1.
