# Supplementary material for: Kinetic Characterization of Cerium and Gallium Ions as Inhibitors of Cysteine Cathepsins L, K, and S
Source: Int J Mol Sci. 2022 Aug 12;23(16):8993. doi: 10.3390/ijms23168993 (PMC9409168; doi:10.3390/ijms23168993)
Supplement: Supplementary file 1 [file ijms-23-08993-s001.zip › ijms-1847739-supplementary.pdf]

Kinetic Characterization of Cerium and Gallium Ions as Inhibitors of Cysteine Cathepsins L, K, and S

Marko Novinec , Primož Bembič, Milica Janković, Marija Kisilak, Jakob Kljun and Iztok Turel

Supplementary materials

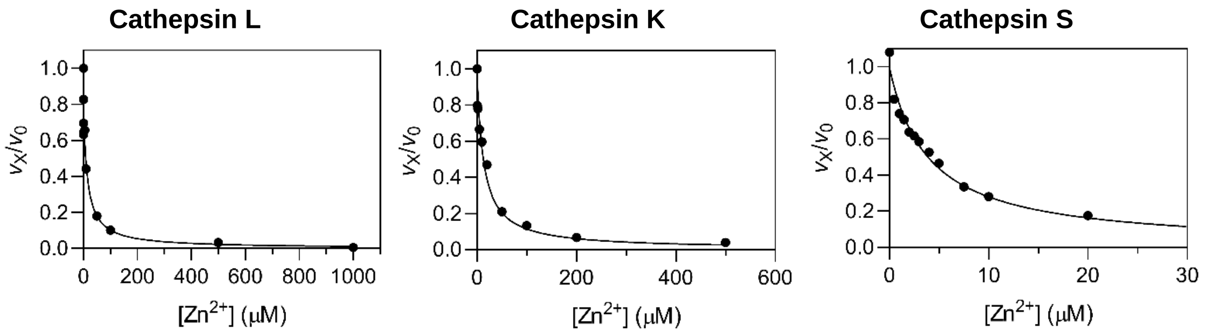

Supplementary Figure S1: Titration of cathepsins L, K and S with Zn^{2+} ions. Titration curves were recorded using synthetic fluorogenic substrates (Z-Leu-Arg-AMC, Z-Phe-Arg-AMC and Z-Val-Val-Arg-AMC for cathepsins L, K and S, respectively) at 25 °C in 0.1 M sodium acetate buffer pH 5.5. σ is equal to $[S]/K_m$.

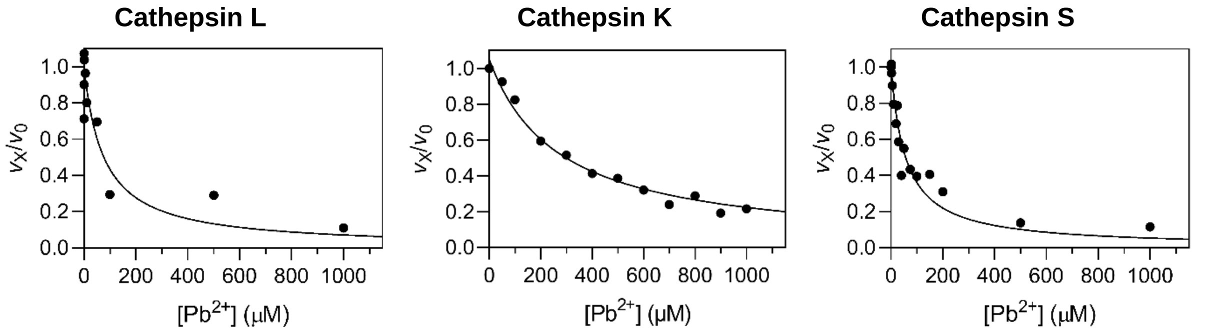

Supplementary Figure S2: Titration of cathepsins L, K and S with Pb^{2+} ions. Titration curves were recorded using synthetic fluorogenic substrates (Z-Leu-Arg-AMC, Z-Phe-Arg-AMC and Z-Val-Val-Arg-AMC for cathepsins L, K and S, respectively) at 25 °C in 0.1 M sodium acetate buffer pH 5.5. σ is equal to $[S]/K_m$.
